# Associations Between Parenting Practices and Peer Pressure Among Adolescents: The Mediating Role of Autonomy and Relatedness

**DOI:** 10.1007/s10935-025-00867-6

**Published:** 2025-07-04

**Authors:** M. A. Crespo-López, I. M. Koning

**Affiliations:** Clinical Child and Family Studies, Faculty of Behavioural and Movement Sciences, Vrije Universiteit Amsterdam, 1081 HV Amsterdam, The Netherlands

**Keywords:** Parenting practices, Peer pressure, Autonomy, Relatedness, SEM

## Abstract

Parents influence their children’s social development, including their susceptibility to peer pressure. Both parenting practices and peer pressure are associated with adolescents' basic needs for autonomy and relatedness, essential for healthy development. However, the mechanisms underlying these relationships require further exploration. Grounded in Self-Determination Theory, this study addresses the following questions: (1) How do different parenting practices (parental support, psychological control, and behavioral control) affect adolescents’ autonomy and relatedness? (2) Do autonomy and relatedness mediate the relationship between parenting practices and susceptibility to peer pressure? (3) Does gender moderate the association between parenting practices and susceptibility to peer pressure? Structural Equation Modelling (SEM) was used to analyse data from 2156 Dutch adolescents (*M*age = 14.67, *SD* = 1.33; 1129 girls). Results indicate that high psychological control—a parenting practice involving guilt induction and love withdrawal—is associated with higher susceptibility to peer pressure, while high parental support is associated with reduced susceptibility. Behavioral control showed no direct effect on peer pressure. Psychological control negatively impacted autonomy and relatedness, while behavioral control and support positively affected relatedness and negatively affected autonomy. Autonomy and relatedness mediated the relationship between psychological control and susceptibility to peer pressure, and between parental support and susceptibility to peer pressure. Gender moderated the relationship between psychological control and susceptibility to peer pressure, with boys showing a stronger association. These findings highlight the importance of supportive parenting in fostering adolescents' autonomy and relatedness, ultimately reducing their susceptibility to peer pressure. They offer valuable insights for developing effective parenting programs tailored to adolescents' needs, incorporating a gender-sensitive approach.

## Introduction

Entering adolescence, individuals undergo complex psychosocial changes and engage in identity exploration through experimentation (Arnett, 2007) and group affiliations (Albarello et al., 2018). During this developmental stage, peers become the primary social reference group, exerting considerable influence on attitudes and behaviors (Rubin et al., 2008). As a result, adolescents are particularly vulnerable to peer influence, more so than individuals at other life stages (Laursen & Veenstra, 2021). Peer pressure, a specific form of peer influence, refers to the subjective or actual experience of being encouraged or compelled by peers to act in accordance with certain guidelines of the peer group (Santor et al., 2000), and can negatively impact well-being (Hoeve et al., 2009; McMillan et al., 2018). It is considered a social factor that increases adolescents’ inclination to engage in risk-taking behaviors (Crosnoe & McNeely, 2008), including substance use (Allen et al., 2006; Hoffman et al., 2007; Leung et al., 2014), sexual risk behavior (Widman et al., 2016), relational aggression (Schad et al., 2008), and delinquent behavior (Sullivan, 2006). Given adolescents' heightened vulnerability to peer influence during this developmental stage, and the well-established association between peer pressure and risk behaviors, it is crucial to identify factors that can reduce their vulnerability to peer pressure.

One such factor is parenting, as research shows that parents play a significant role in shaping adolescents' ability to navigate peer pressure (Bámaca & Umaña-Taylor, 2006; Macuka, 2008). Various parenting practices, such as parental control, which encompasses psychological control (PC) and behavioral control (BC), as well as parental support (PS), have a direct effect on adolescents' susceptibility to peer pressure (Chan & Chan, 2013; Lebedina-Manzoni & Ricijas, 2013). PC involves invalidating and manipulating children's emotional experiences, using tactics such as guilt induction and emotional withdrawal (Barber, 1996; Barber et al., 2002). In contrast, BC refers to managing adolescent behavior through supervision, setting limits, and establishing and enforcing household rules (Barber, 1996). Lastly, PS focuses on nurturing the emotional aspects of the parent–child relationship, emphasizing involvement, warmth, and responsiveness (Cummings et al., 2000). Research indicates that PC is associated with increased social withdrawal (Pinquart, 2017) and heightened susceptibility to peer pressure (Chan & Chan, 2013; Lebedina-Manzoni & Ricijas, 2013). In contrast, BC and PS are linked to reduced susceptibility to peer pressure (Chan & Chan, 2013; Lebedina-Manzoni & Ricijas, 2013) and a stronger sense of peer acceptance (Marcone et al., 2018). While the influence of these parenting practices on susceptibility to peer pressure is established, the specific mechanisms through which they operate remain unclear.

### Basic Psychological Needs Theory

While parenting practices directly influence adolescents' susceptibility to peer pressure, they also affect the fulfillment of adolescents' basic psychological needs, which in turn may impact their susceptibility to peer pressure (Chan & Chan, 2013; Costa et al., 2016, 2019; Wei et al., 2022). These needs—autonomy, competence, and relatedness—are central to the Basic Psychological Needs Theory (BPNT), a sub-theory within the broader Self-Determination Theory (SDT; Deci & Ryan, 2000, 2012). Autonomy refers to the inherent desire for self-direction and psychological freedom, whereas competence pertains to the feeling of capability to achieve goals. Relatedness, conversely, encompasses the need for intimate connections and a sense of belonging (Deci & Ryan, 2000, 2008). According to BPNT, satisfying these universal psychological needs is essential for well-being, and parenting practices can either support or thwart these needs (Abidin et al., 2023).

Kagitcibasi’s theory of autonomous-relatedness self emphasizes the centrality of autonomy and relatedness over competence in parent–child relationships, leading scholars to focus on how parenting practices influence particularly these two needs (Fousiani et al., 2016; Ingoglia et al., 2021; Inguglia et al., 2015; Kagitcibasi, 2005). PC can undermine autonomy by pressuring adolescents to conform to specific thoughts or behaviors and weaken relatedness through conditional parental regard (Scharf & Goldner, 2018; Soenens & Vansteenkiste, 2010). In contrast, supportive parents effectively promote their children's basic autonomy and relatedness (Inguglia et al., 2015). Similarly, BC, through supervision and structure, contributes positively to these needs (Costa et al., 2019; Inguglia et al., 2015). Therefore, parenting practices play a crucial role in shaping adolescents' autonomy and relatedness, with PS and BC promoting these needs, while PC may undermine them.

Beyond the well-established associations between parenting practices and autonomy and relatedness, these needs have also been associated with peer pressure in adolescents. According to SDT, feeling of environmental pressure diminishes an adolescent's sense of genuine choice, which contributes to the frustration of their basic needs (Neighbors et al., 2004). Furthermore, adolescents with unmet autonomy and relational needs may struggle to connect with peers, navigate interpersonal conflicts effectively, and might adopt a passive role in peer interactions (Allen et al., 2007; Bámaca & Umaña-Taylor, 2006; Hentges & Wang, 2018; Inguglia et al., 2015). While the direct connections between parenting practices, basic needs and peer pressure are established, the precise underlying mechanisms of these associations remain unclear.

Recent studies have explored how parenting practices affect adolescents’ well-being through the mediating effect of basic needs (Ahmad et al., 2013; Chan & Chan, 2013; Costa et al., 2016, 2019; Fousiani et al., 2016; Wei et al., 2022). Specifically, these studies found that satisfaction of basic needs mediated the relationship between PS and well-being, and their frustration mediated the relationship between PC and internalizing (Chan & Chan, 2013; Costa et al., 2016, 2019) and externalizing symptoms (Ahmad et al., 2013; Wei et al., 2022). However, no studies have yet explored how these processes affect susceptibility to peer pressure. This study aims to fill that gap by examining whether autonomy and relatedness mediate the relationship between parenting practices and peer pressure in adolescents.

Research suggests that boys generally report higher levels of hostility and parental control compared to girls (Ortega et al., 2023; Rodríguez et al., 2009). Moreover, boys appear more sensitive to negative parenting practices (Roman et al., 2024), and show an increased tendency to externalizing disorders under PC (León-del-Barco et al., 2019). Therefore, it is expected that parenting practices will have a greater impact on boys' susceptibility to peer pressure than on girls.

### Purpose of the Present Study

To our knowledge, this is the first study to examine how parenting practices (PC, BC, and PS) are linked to susceptibility to peer pressure through the mediating role of basic needs. Based on SDT, this study seeks to clarify these mechanisms in a sample of Dutch adolescents.

Drawing on previous research, we hypothesize the following: (1) higher levels of PC will be associated with lower levels of autonomy and relatedness, while higher levels of BC and PS will be associated with higher levels of these two needs; (2) higher levels of autonomy and relatedness will be associated with lower susceptibility to peer pressure; (3) autonomy and relatedness will mediate the relationship between parenting practices and susceptibility to peer pressure. Specifically, PS and BC will enhance autonomy and relatedness, reducing susceptibility to peer pressure, while PC will hinder these needs, increasing susceptibility.

Additionally, this study explores gender as a moderator in the association between parenting practices and peer pressure. We hypothesize that parenting practices will have a stronger effect on boys’ susceptibility to peer pressure than on girls.

## Method

### Sample

A total of 2166 high-school students, including 1037 men (47.88%), participated in the study. The participants’ ages ranged from 11.75 to 18.86 (*M*age = 14.67, *SD* = 1.33). These participants were enrolled in two Dutch high schools. In terms of their living situation, 1738 (80.2%) reported living with both parents, 363 (16.7%) had divorced parents, and 66 (3%) reported a different living situation. The distribution of students across different educational tracks was as follows: 211 students (9.7%) were in the lower vocational track (“VMBO”), 448 (20.7%) in VMBO theoretical/mixed track, 516 (23.8%) in higher general education (“HAVO”), 189 (8.7%) in VMBO/HAVO, 471 (21.7%) in pre-university education (“VWO”), and 331 (15.3%) in HAVO/VWO.

### Procedure

This cross-sectional study was conducted as part of an ongoing community-based alcohol intervention project in two secondary schools in a municipality in The Netherlands (LEF; Koning et al., 2021). Before data collection, ethical approval was obtained from the Faculty Ethical Review Committee (FETC18-060). Data driven from self-questionnaires were electronically coded and safeguarded in a secure environment, ensuring compliance with the European Union General Data Protection Regulations. All data were pseudonymized when stored, associating a number to each participant, and subsequently de-identified and anonymized for sharing. Only authorized researchers had access to this data.

Given that the participants were underage, informed consent was obtained from their parents or legal guardians. Parents received detailed information about the study’s purpose, the voluntary nature of participation, data privacy, safety measures, and ethical considerations. They were informed of their right to withhold consent for their child’s participation. Additionally, students were assured that their own participation was voluntary and that they could withdraw from the study at any time without consequences. The researchers administered the questionnaires in the classroom, emphasizing that participation was both voluntary and confidential. The research team was available to address any questions from students.

A total of 2166 students agreed to participate in the study. However, 10 students did not complete the entire questionnaire and were therefore excluded from the final sample. As a result, a sample of 2156 adolescents were eligible for analysis.

### Measurements

All the questionnaires were administered in Dutch. First, an ad hoc questionnaire was developed to collect sociodemographic information, including age, gender, school, educational level, and school year. Gender was assessed with the question “Ben je een jongen of een meisje?” (“Are you a boy or a girl?”), with response options “boy” and “girl.” This phrasing reflects gender identity as understood and expressed by the participants, rather than sex assigned at birth. This was followed by five brief self-report measures evaluating parental support, parental psychological control, parental behavioral control, basic needs (autonomy and relatedness), and perceived peer pressure. In this study, all the scales exhibited acceptable to good internal reliability (Table 1).

**Table 1:** Descriptive statistics and correlation analyses

Variable	α	*M*	*SD*	Skew	Kurt	1	2	3	4	5
1. PS	0.89	6.09	1.19	− 1.79	3.42					
2. BC	0.83	3.36	1.06	− 0.23	− 0.83	0.05*				
3. PC	0.82	1.98	0.75	0.70	0.25	− 0.39**	− 0.07**			
4. Auton	0.72	5.06	1.02	− 0.28	− 0.19	− 0.02	− 0.12^**^	− 0.13^**^		
5. Relat	0.72	5.11	1.05	− 0.48	0.12	0.54^**^	0.08^**^	− 0.42^**^	0.01	
6. PPress	0.89	1.68	0.70	1.11	1.59	− 0.16^**^	− 0.01	0.25^**^	− 0.18^**^	− 0.23^**^

*PS* Parental support, *BC* Behavioural control, *PC* Psychological control, *Auton* Autonomy, *Relat* Relatedness, *PPress* Peer pressure. Scores of the diagonal = Cronbach’s coefficient alpha. **p < 0.01; *p < 0.05

#### Parental support

Parental support was measured using the family support subscale from the Multidimensional Scale of Perceived Social Support (MSPSS; Zimet et al., 1988). The MSPSS is a widely used self-report instrument designed to measure the perceived availability of support from three sources: family, friends, and significant others. An example item is “I get the emotional support I need at home.” Participants responded to four items on a 7-point Likert-type scale ranging from 1 (*Strongly Disagree*) to 7 (*Strongly Agree*). A higher average score indicated a higher level of parental support. The MSPSS has demonstrated strong reliability and validity in adolescent populations (Canty-Mitchell & Zimet, 2000; Merino-Soto et al., 2022; Trejos-Herrera et al., 2018).

#### Psychological Control

The 8-item Psychological Control Scale-Youth Self-Report (PCS-YSR; Barber, 1996; Barber et al., 2002) was used to assess perceived parental psychological control. An example item is “My parent changes the subject when I have something to say.” Participants responded to the items on a Likert-type scale ranging from 1 (*Not applicable at all*) to 5 (*Totally applicable*). Mean scores were computed, with higher scores representing more psychological control. This scale’s reliability and validity have been consistently supported across diverse cultural contexts (Barber et al., 2012; Habibi Asgargab et al., 2023).

#### Behavioural Control

Parental behavioral control was assessed using the measure reported by Kerr and Stattin (2000). Items focused on parental monitoring and supervision of the adolescent’s behavior (e.g., “Do you need your parents’ permission to stay out late on a weekday evening?”). Responses were given on a 5-point scale ranging from 1 (*Never*) to 5 (*Always*). A higher score indicated more behavioral control. This measure has shown strong psychometric properties in previous studies (Lionetti et al., 2016).

#### Autonomy

Autonomy was measured with a six-item scale. Three items were adapted from the autonomy scale developed by Güngör and Phalet (2011) and validated for both Belgian and Turkish samples (e.g., "I can plan my future without my parent's guidance"; Güngör & Phalet, 2011). The other three items were adapted from a Belgian scale on children’s autonomy (e.g., "I usually find it comforting if my parent chooses in my place what is good for me," reversed item). Participants rated these items on a 7-point Likert-type scale (1 = *Totally disagree*; 7 = *Totally agree*). Mean scores were calculated, with higher scores indicating higher levels of autonomy.

#### Relatedness

Relatedness to parents was measured using a six-item scale adapted from the relatedness scale developed by Güngör and Phalet (2011) (e.g., "My relationship with my parent is an important part of who I am"). Participants rated these items on a 7-point Likert-type scale (1 = *Totally disagree*; 7 = *Totally agree*). Mean scores indicated a stronger sense of relatedness to parents. This scale was chosen based on Coskan’s (2016) findings, which showed it provided optimal conceptual coverage for relatedness in parent-adolescent relationships.

#### Peer Pressure

Susceptibility to peer pressure was measured with a newly developed scale in the SNARE (Social Network Analysis of Risk behavior in Early adolescence) study (Laninga-Wijnen et al., 2023). An example item is, "Some young people do certain things that they normally would not do because otherwise, they will not be a member of their group." The six items could be answered on a Likert-type scale with categories ranging from 1 (*Does not apply to me*) to 5 (*Does often apply to me*). Higher mean scores indicated more susceptibility to peer pressure. A factor analysis (PCA with varimax rotation) was performed on SPSS-27 and confirmed the one-factor structure of the scale, with an eigenvalue of 3.94 and 66% variance explained.

### Statistical Analyses

Descriptive statistics and correlation analyses were initially conducted using SPSS-27 (see Table 1) to provide an overview of the data.

Before testing the theoretical model, the dataset was checked for outliers and adherence to normality assumptions using SPSS-27. Skewness and Kurtosis tests were conducted to evaluate the distribution of the dependent variables, with Kim’s (2013) guidelines applied to identify significant deviations from normality. Specifically, skewness values exceeding ± 2 and kurtosis values exceeding ± 7 were considered indicative of substantial non-normality. This step ensured the appropriateness of the data for Structural Equation Modelling (SEM), which assumes normality for accurate model estimation.

Multicollinearity was also examined by calculating bivariate correlations among all variables, considering correlations above 0.90 indicative of potential multicollinearity issues, following Kline’s (2023) recommendations. This assessment helped ensure the independent variables (parenting practices, autonomy, and relatedness) provided unique information without redundant overlap.

Structural Equation Modelling (SEM) was performed using the R package *lavaan* (Rosseel, 2012), which is particularly suited for testing complex theoretical models that include both direct and indirect effects. SEM was chosen because it allows for the simultaneous testing of multiple relationships, offering a comprehensive understanding of how parenting practices (PS, PC, and BC) influence susceptibility to peer pressure through autonomy and relatedness as mediators. This method also allowed us to evaluate the direct, indirect, and total effects of each independent variable on the dependent variable, making it an appropriate choice for exploring mediation pathways.

Model fit was evaluated using established fit indices in SEM research as indicators of model adequacy: the Chi-square (χ^2^), Comparative Fit Index (CFI), and Tucker-Lewis Index (TLI). Following West et al. (2012), CFI and TLI values greater than 0.95 were considered indicative of a well-fitting model.

To assess mediation effects, bootstrapping with 5000 replication samples was employed to estimate the confidence intervals of both direct and indirect effects, using a 95% bias-corrected confidence interval (Preacher & Hayes, 2008). Bootstrapping was selected as it provides a robust estimate of mediation effects without relying on normality assumptions, which is particularly useful given the complex interactions in the model. A significant total indirect effect would indicate that the independent variables (parenting practices) influence the dependent variable (susceptibility to peer pressure) through the mediating variables (autonomy and relatedness). Specific indirect effects were also examined to determine which mediation pathways were significant, further clarifying the mechanisms linking parenting to peer pressure susceptibility.

Additionally, gender was included in this model as a categorical moderator (0 = boy, 1 = girl), to test whether the direct relationships between parenting practices and peer pressure susceptibility differed by gender. This decision was informed by previous research highlighting gender differences in reported parenting practices (Ortega et al., 2023; Rodríguez et al., 2009) and variations in susceptibility to their effects (León-del-Barco et al., 2019).

Statistical significance was set at *p* < 0.05 for all analyses, and all reported coefficients are standardized.

## Results

### Descriptive Statistics and Correlations

The descriptive statistics, Cronbach’s alpha values, and correlations for the study variables are presented in Table 1. Correlations showed that parenting practices were significantly related to autonomy and relatedness, except PS and autonomy (*r* = − 0.02, *p* > 0.05). PS was also significantly and positively related to susceptibility to peer pressure (*r* = − 0.16, *p* < 0.01), while PC was negatively related to peer pressure (*r* = 0.25, *p* < 0.01).

### Stepwise Mediation Models

The SEM approach was used to test our mediation models, which was advanced by Baron and Kenny (1986). In line with recommended practices for testing mediation in complex models (MacKinnon et al., 2007), we used a stepwise model-building approach.

First, we estimated a direct effects model (Model 1), in which PC, BC, and PS were entered simultaneously to predict susceptibility to peer pressure. These parenting practices were included together based on their theoretical distinctiveness and because adolescents are typically exposed to all of them in the family context. Estimation of this model, *χ2* = 151.81, *df* = 3, *p* < 0.001, *CFI* = 0.99, *TLI* = 0.99, showed a negative significant path from PS to peer pressure (*β* = − 0.043; *p* < 0.001), a positive significant path from PC to peer pressure (*β* = 0.210; *p* < 0.001), and a non-significant path from BC to peer pressure (*β* = − 0.018; *p* > 0.05). That is, more parental support and a lower level of psychological control related significantly to lower susceptibility to peer pressure.

Second, we estimated two models, in which the parenting variables predicted each mediator separately: autonomy (Model 2a) and relatedness (Model 2b). Model 2a was statistically significant, *F*(3, 2127) = 24.88, *p* < 0.001, and explained 3.4% of the variance in autonomy. All three parenting practices were significant negative predictors of autonomy.

PC had the strongest effect (*β* = − 0.160,* p* < 0.001), followed by BC (*β* = − 0.096, *p* < 0.001), and PS (β = − 0.079, *p* < 0.001). These results indicate that higher levels of psychological and behavioral control, as well as parental support, are associated with decreased autonomy. Model 2b was also statistically significant, *F*(3, 2127) = 371.47, *p* < 0.001, and explained 34.4% of the variance. All three predictors were statistically significant. PS had a strong positive effect (*β* = 0.434, *p* < 0.001), PC had a moderate negative effect (*β* = − 0.251, *p* < 0.001), and BC had a small positive effect (*β* = 0.075, *p* < 0.001). Thus, parental support appears to be the strongest contributor to adolescents’ sense of relatedness, while psychological control undermines it.

Third, we tested a model (Model 3) in which autonomy and relatedness predicted susceptibility to peer pressure. This model was also statistically significant, *F*(2, 2128) = 100.58, *p* < 0.001, and explained 8.6% of the variance in peer pressure. Both autonomy (*β* = − 0.180, *p* < 0.001) and relatedness (*β* = − 0.230, *p* < 0.001) were negatively associated with susceptibility to peer pressure, indicating that higher levels of either mediator predict lower susceptibility.

**Fig. 1:**
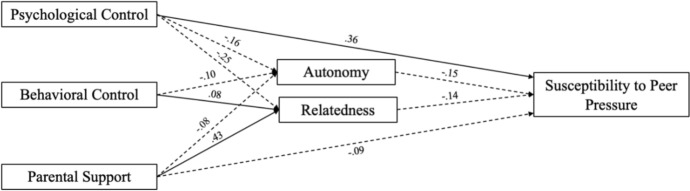
Graphical representation of the structural equation model depicting significant relationships. The coefficients shown are standardized path coefficients (std.all). The dotted lines represent negative relations. Non-significant paths are not reported

Finally, we tested our full mediation model (Model 4), in which parenting practices were related directly and indirectly to peer pressure through autonomy and relatedness and moderated by gender (Fig. 1). The model was estimated using Maximum Likelihood (ML) and converged normally after 4 iterations. The chi-square test of model fit was significant, *χ*^2^(6) = 40.687, *p* < 0.001, indicating some degree of misfit between the model and the observed data. However, the chi-square test is highly sensitive to sample size and should be interpreted in conjunction with alternative fit indices. Other indices suggest that the hypothesized model provided an acceptable to good fit to the data: *CFI* = 0.974, *TLI* = 0.909.

### Direct Effects Full Model

In line with the previous model, the direct effect (*c*’) of PS on peer pressure was negative and significant (*β* = − 0.061, *p* < 0.05). This indicates that higher parental support is associated with lower susceptibility to peer pressure. PC showed a significant positive direct effect on peer pressure susceptibility (*β* = 0.225, *p* < 0.001), suggesting that higher levels of psychological control are related to greater susceptibility to peer pressure. Finally, the direct effect of BC on peer pressure was not significant (*β* = 0.012, *p* = 0.676), indicating that behavioral control does not significantly predict susceptibility to peer pressure.

### Indirect Effects Full Model

The coefficients *a* reveal the influence of the independent variables as predictors of the mediator variables. The coefficients for all variables were significant (*p* < 0.001). Specifically, more PS was significantly associated with higher levels of relatedness (*β* = 0.433, *p* < 0.001) but, interestingly, lower levels of autonomy (*β* = − 0.080, *p* < 0.001). Similarly, higher PC was significantly related to lower levels of both autonomy (*β* = − 0.159, *p* < 0.001) and relatedness (*β* = − 0.252, *p* < 0.001). Finally, BC negatively predicted autonomy (*β* = − 0.100, *p* < 0.001) and positively predicted relatedness (*β* = 0.078, *p* < 0.001), suggesting that more behavioral control is associated with lower autonomy but higher relatedness.

The coefficients *b* estimate the effects of each mediator on the dependent variable. The effect of autonomy on peer pressure was negative and significant (*β* = − 0.150, *p* < 0.001), as was the effect of relatedness (*β* = − 0.137, *p* < 0.001). This indicates that higher levels of autonomy and relatedness are associated with lower susceptibility to peer pressure.

Figure 1 shows a visual representation of the model, specifically the significant paths and their standardized beta coefficients.

The mediation analysis examined the specific indirect effects of PS, PC, and BC on susceptibility to peer pressure through autonomy and relatedness. These indirect effects represent the product (a*b) of the path coefficients for the relationships between independent variables and each mediator (*a*) and the dependent variable (*b*).

The indirect effect of PS on peer pressure via autonomy was positive and significant (*β* = 0.012, *p* < 0.05), indicating that autonomy positively mediated the relationship between parental support and susceptibility to peer pressure. In addition, the indirect effect through relatedness was negative and significant (*β* = − 0.059, *p* < 0.001), suggesting that relatedness also mediated this relationship, but negatively. Together, these findings show that while autonomy increases the influence of parental support on susceptibility to peer pressure, relatedness decreases it.

For PC, the indirect effects through both mediators were positive and significant. High PC related to high susceptibility to peer pressure via lower autonomy (*β* = 0.024, *p* < 0.001) and lower relatedness (*β* = 0.034, *p* < 0.001).

Finally, the indirect effects for BC were mixed. The indirect effect of BC on peer pressure via autonomy was positive and significant (*β* = 0.015, *p* < 0.05), while the effect via relatedness was negative and significant (*β* = − 0.011, *p* = 0.001). This shows high BC related to high susceptibility to peer pressure via lower autonomy (β = 0.010, *p* < 0.05), and to low susceptibility to peer pressure via higher relatedness (β = − 0.007, *p* = 0.001).

The total indirect effects were calculated by summing the indirect effects of each parenting practice on peer pressure through each mediator. This effect indicates if, collectively, the mediators explain a substantial portion of the relationship between the independent variable and the dependent variable. For PS, the total indirect effect was negative and significant (*β* = − 0.047, *p* < 0.001). PC showed a positive and significant total indirect effect *(β* = 0.058, *p* < 0.001). Finally, the total indirect effect for BC was non-significant (*β* = 0.004, *p* = 0.364).

Table 2 shows the path estimates, standard errors, and confidence intervals for the mediation model.

**Table 2:** Path estimates, SEs, 95% CIs for partial mediation models, and fully standardized coefficients

Effects	Unstandardized estimates	SE	Lower bound (BC) 95% CI	Upper bound (BC) 95% CI	β stand. coeff
*Direct effect*					
PS → autonomy	− 0.068**	0.020	− 0.107	− 0.029	− 0.080
PS → relatedness	0.381**	0.017	0.348	0.414	0.433
PC → autonomy	− 0.218**	0.032	− 0.280	− 0.156	− 0.159
PC → relatedness	− 0.356**	0.027	− 0.409	− 0.303	− 0.252
BC → autonomy	− 0.096**	0.020	− 0.137	− 0.056	− 0.100
BC → relatedness	0.077**	0.017	0.043	0.111	0.078
PS → peer pressure	− 0.035*	0.016	− 0.067	− 0.003	− 0.061
PC → peer pressure	0.211**	0.027	0.157	0.265	0.225
BC → peer pressure	0.008	0.019	− 0.029	0.045	0.012
Autonomy → peer pressure	− 0.103**	0.014	− 0.130	− 0.075	− 0.150
Relatedness → peer pressure	− 0.091**	0.016	− 0.123	− 0.059	− 0.137
*Indirect effect *via* autonomy*					
PS → peer pressure	0.007*	0.002	0.003	0.011	0.012
PC → peer pressure	0.022**	0.004	0.014	0.031	0.024
BC → peer pressure	0.010**	0.002	0.005	0.015	0.015
*Indirect effect *via* relatedness*					
PS → peer pressure	− 0.035**	0.006	− 0.047	− 0.022	− 0.059
PC → peer pressure	0.032**	0.006	0.020	0.045	0.034
BC → peer pressure	− 0.007**	0.002	− 0.011	− 0.003	− 0.011

B-SE Bootstrapped standards errors, BC 95% CI Bias corrected-confidence interval. **p < 0.01; *p < 0.05

The total effects represent the combined influence of the direct and total indirect effects from each parenting practice (PS, PC, and BC) on susceptibility to peer pressure, providing an assessment of the overall influence of an independent variable on the dependent variable, accounting for both direct and mediated paths. The total effects revealed different patterns in our mediation model. PS showed a significant negative total effect on peer pressure (*β* = − 0.108), indicating that higher levels of parental support are associated with reduced susceptibility to peer pressure. In contrast, PC demonstrated a strong positive total effect (*β* = 0.283), suggesting that greater psychological control exerted by parents increases susceptibility to peer pressure. Behavioral control, however, showed a negligible total effect on peer pressure (*β* = 0.016), indicating that its overall influence on susceptibility to peer pressure is minimal.

### Moderation by Gender

Finally, gender was examined as a moderator in the relationship between parenting practices and peer pressure. The results indicated that gender significantly moderated the relationship between PC and susceptibility to peer pressure (*β* = − 0.193, *p* < 0.001), suggesting that the effect of psychological control on susceptibility to peer pressure is stronger for boys than girls. However, gender did not significantly moderate the relationship between PS and susceptibility to peer pressure (*β* = 0.081, *p* = 0.200) or between BC and susceptibility to peer pressure (*β* = − 0.074, *p* = 0.289).

## Discussion

This study contributes to the growing literature on Self-Determination Theory (SDT) and Basic Psychological Needs Theory (BPNT) by exploring how autonomy and relatedness mediated the relationship between parenting practices and susceptibility to peer pressure in Dutch adolescents. The findings align with previous research, showing that higher psychological control is associated with higher susceptibility to peer pressure, while higher parental support is associated with reduced susceptibility. Interestingly, behavioral control did not show a direct relationship with peer pressure. Furthermore, autonomy and relatedness strengthened the relationship between psychological control and susceptibility to peer pressure. Conversely, the relationship between parental support and susceptibility to peer pressure was weakened through a combination of higher relatedness and lower autonomy.

In line with our first hypotheses, more parental psychological control was related to higher susceptibility to peer pressure, and more parental support predicted decreased susceptibility Among the parenting variables, psychological control consistently emerged as the strongest risk factor. This is consistent with previous research (Chan & Chan, 2013; Lebedina-Manzoni & Ricijas, 2013), which suggests that adolescents who experience more psychological control are more likely to seek more support and guidance from their peers, lacking the autonomy necessary for independent judgment and, consequently, becoming more susceptible to peer pressure. In contrast, adolescents with supportive parents may develop stronger parental bonds, which help reduce their susceptibility to external influences (Chan & Chan, 2013) like peer pressure.

However, we found no significant relationship between behavioral control and susceptibility to peer pressure when tested without mediators, in contrast to previous studies that also assessed the direct relationship and reported a significant negative association (Chan & Chan, 2013; Lebedina-Manzoni & Ricijas, 2013). One possible explanation is that during adolescence, teens who perceive their parents as more controlling may prioritize peers norms and standards even more over parental rules and limits (Laursen & Veenstra, 2021). This shift could reflect the deidentification process, wherein adolescents seek to establish their own identity by embracing behaviors and attitudes that differentiate them from their parents (Koepke & Denissen, 2012). Also, research suggests that the effects of behavioral control may differ depending on whether the mother or father exerts control (Chan & Chan, 2013), indicating that the impact of behavioral may be more complex than initially anticipated. Another possible explanation is that adolescents may not always experience behavioral control in a negative way. For instance, boundary setting can be perceived as a sign of genuine care and concern for the teenager’s safety and overall development. Last, different from previous studies that examined behavioral control in isolation, the current study modeled it alongside psychological control and parental support, which may have influenced its observed effects on susceptibility to peer pressure.

Regarding our second set of hypotheses, higher levels of psychological control were associated with lower levels of autonomy and relatedness with parents. Also, higher levels of behavioral control and parental support related to higher levels of relatedness. This supports the idea that psychological control undermines the adolescents’ ability to develop autonomy, while parental conditional regard, which is characteristic of psychological controlling parents, undermines adolescents' ability to form relatedness, thereby weakening the parent–child bond (Scharf & Goldner, 2018; Soenens & Vansteenkiste, 2010). In contrast, parents being supportive towards their adolescent child may effectively promote a strong sense of relatedness (Inguglia et al., 2015). Therefore, these findings highlight the importance of avoiding intrusive parenting practices, such as psychological control, which can weaken or diminish autonomy and negatively affect the emotional bond with their children. On the other hand, behavioral control and parental support seem to be effective strategies for fostering a strong connection and meeting adolescents’ basic needs.

Contrary to expectations and existing literature (Costa et al., 2019; Inguglia et al., 2015), higher levels of behavioral control and parental support were associated with lower autonomy. It is possible that adolescents in the current study may perceive their parents as too controlling or excessively involved. As a result, parental behaviors such as supervision or setting limits may be experienced as overly intrusive, thereby limiting adolescents’ sense of personal agency (Kakihara & Tilton-Weaver, 2009). Furthermore, overly supportive or over-involved parents may unintentionally inhibit the adolescents’ use of their skills and knowledge, resulting in a reduced sense of autonomy (Fingerman et al., 2012). Another explanation could be that the type and manner of parental support affect autonomy differently. Support offered in a way that respects the teenager's autonomy and promotes self-direction may enhance empowerment, while overly directive support that implies that the teenager is incapable of handling things independently may undermine their autonomy (Soenens et al., 2007). This suggests that specific forms and degrees of parental support and behavioral control can either enhance or undermine adolescents' autonomy.

Our findings partially supported our mediation hypotheses. Adolescents with more supportive parents reported lower susceptibility to peer pressure through a combination of higher relatedness and lower autonomy, an unexpected finding that has been elaborated on earlier in this discussion. In contrast, those with parents who exert psychological control showed increased susceptibility via diminished autonomy and relatedness. These results underscore the notion that parents can shape adolescents’ psychosocial development and the influence of peers by supporting their needs for autonomy and relatedness (Bülow et al., 2022; Chan & Chan, 2013; Soenens et al., 2017). Conversely, adolescents who do not have their needs for autonomy and connection met by their parents may turn to their peers for guidance and validation, increasing their susceptibility to peer pressure (Hentges & Wang, 2018; Inguglia et al., 2015).

Finally, the results also partially supported our moderation hypothesis regarding gender differences. The effect of psychological control on susceptibility to peer pressure was stronger for boys than for girls. This is consistent with prior research suggesting that boys are more affected by negative parenting practices, such as psychological control (León-del-Barco et al., 2019; Roman et al., 2024), which could contribute to an increased susceptibility to peer pressure and subsequent higher levels of risk behavior (Hentges & Wang, 2018).

### Implications

Our findings offer valuable insights into how parenting practices influence adolescents’ social development and pave the way for future research to explore context-specific strategies that promote positive adolescent growth. However, it is important to interpret these results with caution, as the observed effect sizes were generally small.

Nonetheless, the findings highlight key recommendations for effective parenting prevention and intervention programs: the importance of raising awareness about the negative effects of psychological control and actively discouraging parents from using such tactics (Costa et al., 2016; Soenens & Vansteenkiste, 2010). Additionally, the results suggest that parental support plays a more important role in reducing adolescents’ susceptibility to peer pressure than previously recognized. Therefore, parenting programs should not only discourage harmful practices like psychological control but also adopt a positive psychology approach. This could involve focusing on enhancing parental strengths and promoting factors that contribute to adolescents’ well-being, as well as emphasizing the importance of fostering a healthy and supportive parent–child relationship.

### Limitations

This study has some limitations that can be addressed in future research. Firstly, the cross-sectional nature of the data prevents us from determining the causal order of the variables examined. Future longitudinal studies would help clarify how these relationships evolve over time and whether parenting practices influence basic needs and subsequent susceptibility to peer pressure or vice versa (Deci & Ryan, 2012).

Secondly, although the associations found were statistically significant, their effect sizes were small. This means that the practical impact of these findings may be limited. Future research should explore the circumstances under which these relationships might have a stronger influence on adolescents' development. It would also be useful to explore other potential mediators, such as self-control, might influence parenting practices' effect on peer pressure (Meldrum et al., 2013). In addition, age could be an important moderator, as it has been found that autonomy and relatedness carry varying significance and roles across adolescence and emerging adulthood (Inguglia et al., 2015).

Third, a significant limitation of this study is the lack of exploration into how the effects of parenting practices on adolescents’ susceptibility to peer pressure may differ across cultural contexts and parental roles (e.g., mothers vs. fathers) (Chan & Chan, 2013). Parenting styles vary widely based on cultural norms, values, and beliefs about the legitimacy of parental authority in particular situations, which may shape adolescents' experiences and interpretations of parental behavior (Lansford, 2022). However, recent research suggests that certain aspects of parenting practices, such as providing warmth and autonomy support, may universally benefit adolescent development, transcending cultural differences (Bülow et al., 2022).

Lastly, this study focused on peer pressure as a negative influence, but peers can also provide a protective influence by encouraging positive behaviors and reducing engagement in risk-taking behaviors (Thurow et al., 2020). Future research could explore how different types of peer influence interact with parenting practices.

## Conclusion

The findings support a mediation model in which parenting practices influence adolescents’ susceptibility to peer pressure through their effects on autonomy and relatedness. High psychological control was associated with increased susceptibility, while high parental support was linked to reduced susceptibility. Behavioral control did not show a direct effect, suggesting that its impact may depend on how adolescents interpret this practice, either as controlling or as an expression of care and protection, or depending on the broader parenting context. Autonomy and relatedness mediated these associations in different ways. Lower autonomy and relatedness partially explained the association between high psychological control and increased susceptibility to peer pressure. The protective role of parental support was mediated by higher relatedness, while its association with lower autonomy may reflect adolescents’ perception of closeness as limiting their independence. Gender moderated the relationship between psychological control and susceptibility to peer pressure, with boys showing a stronger association.

Although some effect sizes were modest, these findings support the core principles of Basic Psychological Needs Theory, showing that adolescents’ susceptibility to peer pressure is shaped by the extent to which their basic needs for autonomy and relatedness are supported or undermined by parenting practices. Future research should explore these patterns over time, across cultural contexts and types of family, and in relation to both negative and positive peer influences.
